# Mattress-Based Non-Influencing Sleep Apnea Monitoring System

**DOI:** 10.3390/s23073675

**Published:** 2023-04-01

**Authors:** Pengjia Qi, Shuaikui Gong, Nan Jiang, Yanyun Dai, Jiafeng Yang, Lurong Jiang, Jijun Tong

**Affiliations:** School of Information Science and Engineering, Zhejiang Sci-Tech University, Hangzhou 310018, China

**Keywords:** sleep apnea-hypopnea syndrome (SAHS), ballistocardiogram (BCG), respiratory signal, wavelet transform (WT), waveform similarity

## Abstract

A mattress-type non-influencing sleep apnea monitoring system was designed to detect sleep apnea-hypopnea syndrome (SAHS). The pressure signals generated during sleep on the mattress were collected, and ballistocardiogram (BCG) and respiratory signals were extracted from the original signals. In the experiment, wavelet transform (WT) was used to reduce noise and decompose and reconstruct the signal to eliminate the influence of interference noise, which can directly and accurately separate the BCG signal and respiratory signal. In feature extraction, based on the five features commonly used in SAHS, an innovative respiratory waveform similarity feature was proposed in this work for the first time. In the SAHS detection, the binomial logistic regression was used to determine the sleep apnea symptoms in the signal segment. Simulation and experimental results showed that the device, algorithm, and system designed in this work were effective methods to detect, diagnose, and assist the diagnosis of SAHS.

## 1. Introduction

Sleep apnea-hypopnea syndrome (SAHS) is one of the most common and serious sleep problems. Its prevalence is increasing yearly, seriously endangering human health. Clinical signs of SAHS are usually defined as more than 30 recurring episodes of apnea during a 7 h sleep cycle, or an average of more than 5 episodes of apnea and hypopnea per hour during sleep [1,2,3]. However, these sleep symptoms can be difficult to detect because the patient is unconscious during sleep. The above factors directly lead to the low diagnosis rate of SAHS. At the same time, long-term undiagnosed SAHS will lead to more complications, such as the damage of multiple organs, for example, cardiovascular and cerebrovascular organs, increasing the risk of disease [4,5,6]. Polysomnography (PSG) evolved from electroencephalography (EEG) and is currently the internationally recognized gold standard for the diagnosis of SAHS [7,8,9,10]. The PSG can simultaneously detect multiple physiological signals and provide accurate diagnosis evidence to doctors. However, in the application process of PSG, patients need to wear various sensors, which will bring a certain physical and psychological burden to patients and affect the quality of their sleep. In addition, PSG is expensive and difficult to promote to all patients. Therefore, the development of a new diagnostic method can help doctors to improve the diagnostic rate of SAHS, which is of great significance for early detection and diagnosis.

In recent years, some new indirect detection methods, such as smart watches, wearable bracelets, and other sleep tracking devices, have been utilized to detect the sleep state by the amplitude and frequency of human movements and calculate the apnea from the collected cardiac signals [11,12,13]. These devices can help patients to improve their quality of life, extend independent living, and reduce the time necessary for medical staff and medical costs. However, respiratory conditions cannot be directly monitored, and the resulting error is usually relatively high, which does not meet the standards of independent devices for healthcare applications [14,15]. Therefore, a mattress-based non-affecting sleep apnea monitoring system was proposed in this work. First, pressure sensors in the mattress were used to pick up signals generated by body movements during sleep and breathing. Then, the wavelet threshold algorithm was used to separate and de-noise the signals, effectively filter the interference noise of the signals, and reconstruct the effective respiratory and ballistocardiogram (BCG) signals. Then, the logistic regression model of apnea was established to extract the characteristic parameters. Finally, the reliable monitoring of sleep apnea was realized.

## 2. Methods

### 2.1. System Composition and Signal

**Figure 1:**
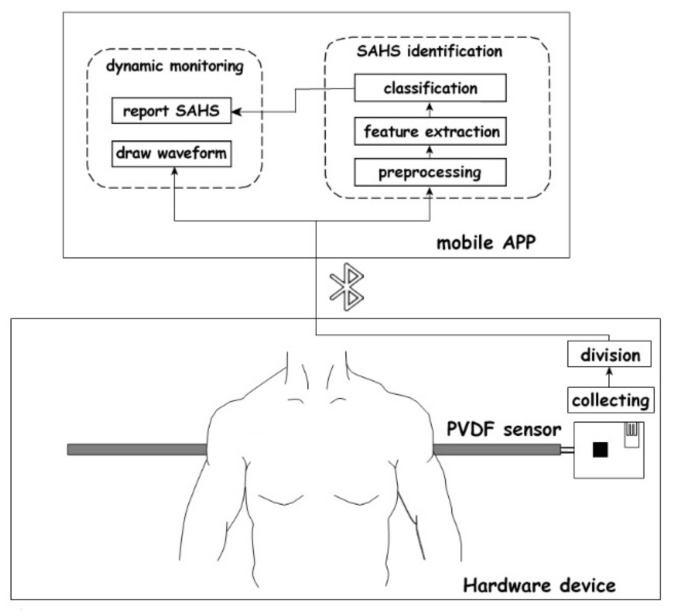
Diagram of mattress-based non-influencing sleep apnea monitoring system.

The diagram of a mattress-based non-influencing sleep apnea monitoring system is shown in Figure 1. The whole system consisted of a hardware device and a mobile application (APP). In the hardware device, a polyvinylidene difluoride (PVDF) sensor was used to collect physiological signals such as heartbeat and respiration, and then sent these signals to the APP via Bluetooth. Then, further analysis, characteristic parameter calculation, and SAHS identification of the received signals were realized in the mobile APP.

PVDF is a typical high-strength polymer piezoelectric material based on the piezoelectric effect, which can be used for pressure signal acquisition. When external forces are applied to the surface of the material, the material will deform, and polarization will occur within the material; then, voltage signals will be generated on the surface of the material. However, when the external force is eliminated, the voltage signal will disappear.

**Figure 2:**
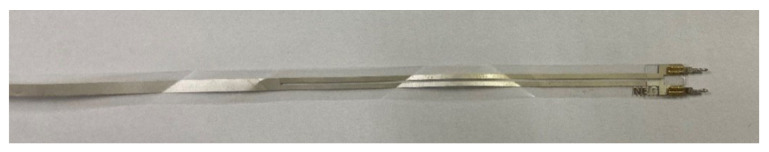
Physical picture of PVDF sensor.

The PVDF sensor used in this work was 800 mm in length, with the capacitance range of 5.49 nF to 9.25 nF, and the sampling rate was set at 50 Hz (Figure 2). The sensor was placed under the mattress or bed sheet to collect the dynamic pressure generated by the human body during sleep in a non-influencing manner, and then obtained physiological signals such as respiration and BCG. This method showed high sensitivity and was suitable for practical application in sleep monitoring.

The respiratory signal is the periodic expansion and contraction of the chest during sleep, with a frequency of about 0.2 to 0.4 Hz [16]. The respiration waveform has a relatively intuitive periodicity, reflecting the process of breathing and thoracic cavity activity. The complete cycle of the respiratory signal waveform should include the process of exhalation and inhalation. From beginning to end, the respiratory waveform corresponds to the movement of the chest as it contracts and relaxes. After being collected by the pressure sensor, the breathing movement is morphologically expressed as the relationship of the voltage converted by the vibration pressure with time.

**Figure 3:**
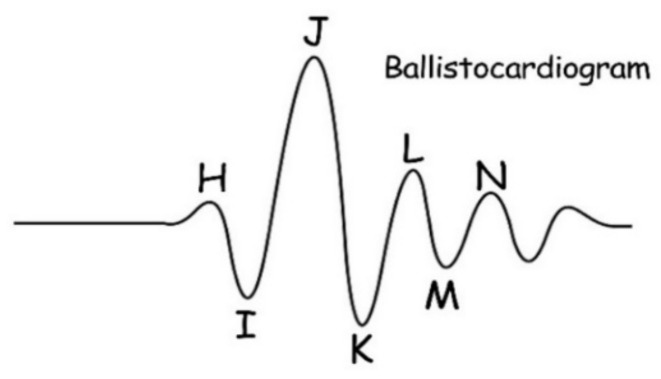
An example of BCG waveform.

BCG is a signal indicating the mechanical activity of the heart. It is obtained by detecting the vibrations caused by blood pumping from the heart into the artery. The frequency distribution of BCG is 0.5 to 20 Hz [17]. BCG is quite different from an electrocardiogram (ECG) in waveform shape. As shown in Figure 3, a typical BCG signal waveform has 7 wave peaks which are labeled as H, I, J, K, L, M, and N. The H, I, J, and K wave peaks indicate the pressure of blood hitting the aorta and large vessels after heart contraction. The L, M, and N wave peaks indicate the diastolic process of heart. The essence of BCG signal acquisition is the acquisition of pressure signal, which can be considered as a non-stationary signal [18]. BCG has special advantages in some medical applications because it can be made into a mattress without the need for users to wear it, with low cost and sufficient performance [19]. The mattress-based pressure sensor was used to collect the BCG signal instead of the ECG signal in our design. The specific performance comparison between BCG and ECG is shown in Table 1.

**Figure 4:**
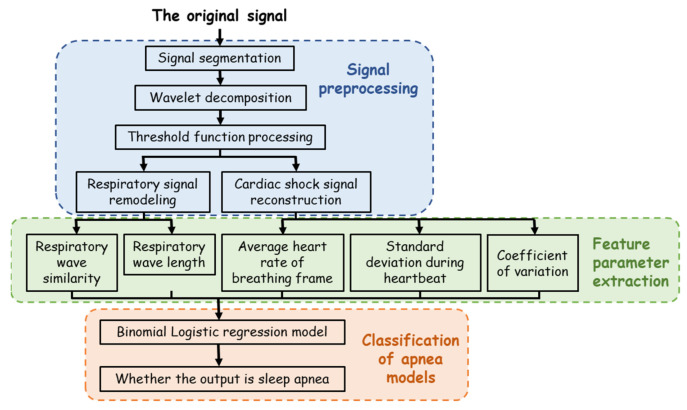
Signal processing algorithm process flow chart.

The SAHS signal processing process is shown in Figure 4, which can be divided into three stages: signal preprocessing, feature parameter extraction, and classification of apnea models. In the preprocessing stage, signal separation and the soft threshold denoising method [20] based on wavelet transform were adopted to effectively eliminate the low-frequency interference, reduce the complexity and calculation of signal extraction, improve the extraction accuracy, and successfully extract the respiratory signal and BCG signal. Then, in the feature extraction stage, the peak searching algorithm based on physiological signal feature optimization was used to extract the feature, which improved the accuracy of index calculation. Finally, in the classification stage, a binary logistic regression model was used to identify SAHS.

### 2.2. Device and Testing

**Figure 5:**
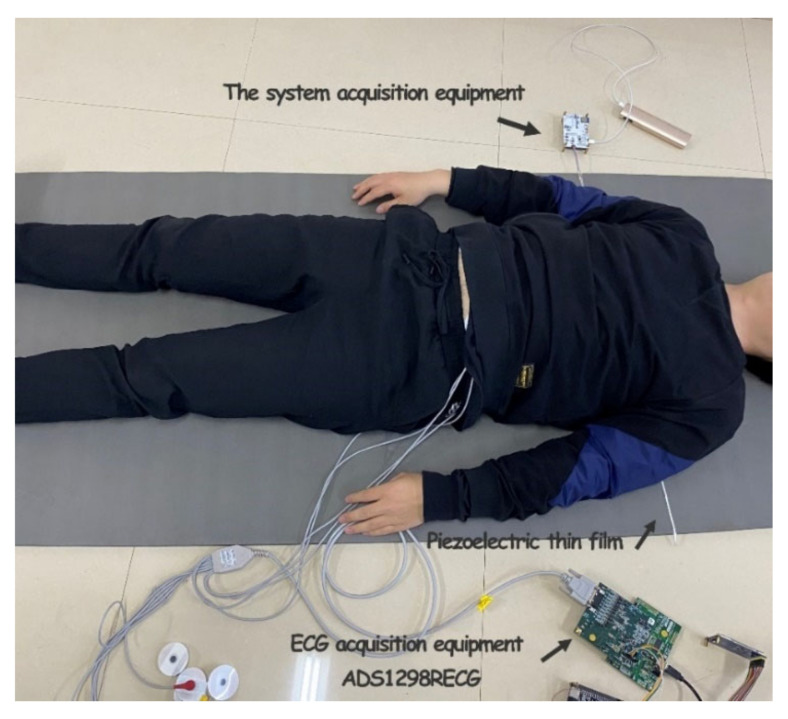
Acquisition system physical picture.

The actual picture of the acquisition system used in the experiment is shown in Figure 5. The acquisition system included hardware acquisition circuits, Android phones, and other environmental items such as mattresses and marketing devices for data comparison. In addition, the acquisition system in this work and TI company’s product ADS1298RECG monitoring device were used to conduct signal acquisition simultaneously.

Twenty adults were selected, including 15 males and 5 females, aged between 22 and 28. All participants expressed their willingness to participate in the test and received instructions during the test. The experiment was carried out on a mattress, the mattress was flattened, and the increased area of the sensing belt was placed at the projection position of the chest when the human body was lying on the bed. The subjects were placed in bed and simulated breathing during normal sleep, randomly simulating post-inhalation apnea and post-exhalation apnea. Each experimenter collected about 50 groups of respiratory frame data, and a total of about 1000 groups of data were taken. Among them, about 800 groups of data from 16 individuals were randomly selected as sample data for model establishment, and 200 groups of data from the remaining 4 individuals were used as experimental verification data.

## 3. Experiments and Results

### 3.1. Signal Preprocessing

During sleep, the pressure signal generated by chest and abdominal vibrations of the human body includes respiratory, BCG, and noise components. The frequency of the respiratory signal is about 0.3 and 2 Hz, and the highest frequency component of the BCG vital sign in the 1–20 Hz frequency range [21]. The noise components include human myoelectric noise, 50 Hz/60 Hz power-line interference, and body motion noise. Therefore, in order to effectively preserve the respiratory and cardiac impulse signals and filter out the impact of various interference noises, we adopted a 40 Hz low-pass filter. During the test, although the low-pass filter had attenuated most of the high-frequency noise, there were still some interferences in the collected original signal, such as the interference generated by the body movement and the noise introduced by the PVDF piezoelectric film material when the elastic deformation occurred. The above interference owned a wide frequency distribution range and was close to the frequency of the effective signal, so it was difficult to be eliminated by the hardware filter. Therefore, the signal after hardware filtering still needed to be processed again by the denoising method. Commonly used signal separation and denoising methods mainly include empirical mode decomposition (EMD), digital bandpass filtering, and wavelet transform (WT) [22,23]. Among them, WT is often used in signal time–frequency analysis and processing. The process of wavelet denoising is decomposition, wavelet packet coefficient thresholding, denoising, and reconstruction. Firstly, the appropriate wavelet basis function is selected. Then, the threshold function and threshold are set to process each decomposition layer, and the new wavelet coefficient is obtained. Finally, the new coefficient is used for wavelet reconstruction, and the cleaned signal waveform is obtained after processing. Therefore, we can conclude that the noise removed by wavelet denoising is theoretically Gaussian noise covering the whole frequency band. This work is to make use of the characteristics of wavelet decomposition by layer (frequency band) and improve the wavelet denoising process into hierarchical decomposition by the frequency band, wavelet packet coefficient threshold, denoising, reconstruction, and separation to obtain the denoised respiratory signal and BCG signal. The advantage of this improvement is that the noise can be removed at the same time of separation, which can improve engineering efficiency. The actual collected sleep signals can be expressed as follows:

(1) $$f\left(t\right)=x\left(t\right)+n\left(t\right)$$

where $f\left(t\right)$ is the collected original sleep fusion signal with noise, $x\left(t\right)$ is the clean sleep piezoelectric signal, and $n\left(t\right)$ is the interference noise.

**Figure 6:**
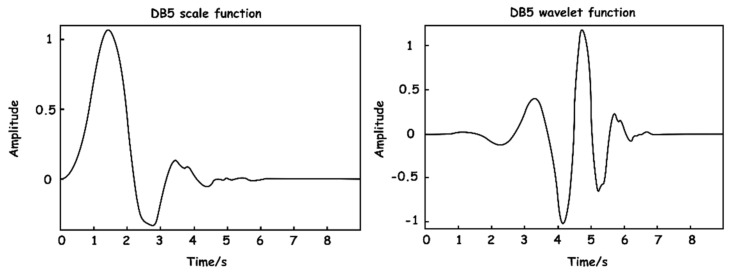
DB5 wavelet base function.

The wavelet basis function selection is a challenge in the actual application of wavelet analysis. Using a variety of different basis functions on the same signal produces a variety of distinct outputs, which are mostly determined by the mathematical and waveform features of the signal to be processed. On the basis of the foregoing principles and the features of the collected pressure signal, the Daubechies transform at level 5 (DB5) function was chosen for signal decomposition in this work. Ingrid Daubechies proposed the DB wavelet function scheme [24]. It has orthogonality, approximate symmetry, a compact support, and a hierarchical structure. Additionally, because the size function of DB5 is comparable to that of a respiratory wave, it may achieve a high vanishing moment, which is favorable to signal breakdown. The scaling function and wavelet function of DB5 are depicted in Figure 6.

**Figure 7:**
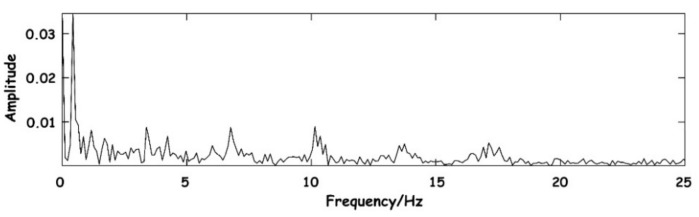
Spectrum diagram of the original signal.

Then, the signal needs to be decomposed into enough layers to ensure that the respiration signal with low relative frequency can be completely preserved and separated from the components of the BCG signal as far as possible in the time domain. The system selects layers through the frequency range of different signal components. The spectrum diagram of the original signal is shown in Figure 7. As can be seen from the figure, signals are mainly concentrated in the low-frequency part, where the DC component is the baseline generated by the level rise of the circuit. A large amount of energy between the 0–1 Hz frequency is floating formed by respiratory signals.

Based on the above analysis, the system finally chose five wavelet decomposition layers to concentrate the noise signal in the same layer as far as possible, and the purpose of denoising can be achieved by removing the layer directly. Table 2 lists the frequency range and signal components of each layer of wavelet decomposition, where the scale is represented by j, the approximate component of the corresponding scale is represented by $D_{j}f$, and the detail component is represented by $A_{j}^{d}$.

**Figure 8:**
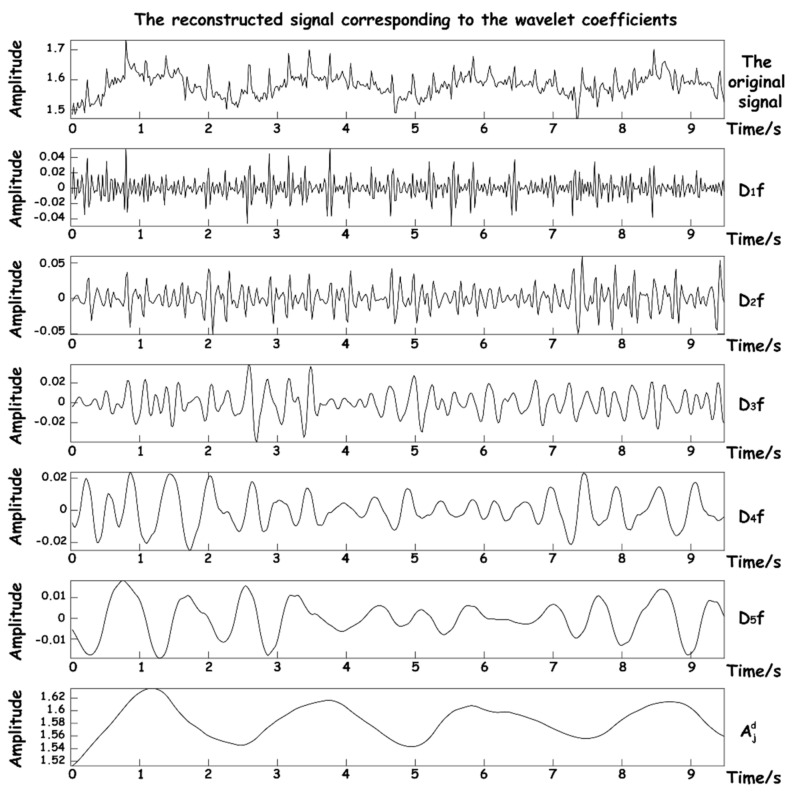
Reconstructed signal graphs at various scales.

In order to show the effect of wavelet decomposition, the operation process of the algorithm in the acquisition was simulated. The sleep signal with a length of 10 s which was collected by the hardware device of this work was input. The quasi-reconstructed signals of each approximate coefficient after wavelet decomposition are shown in Figure 8.

It can be seen that after decomposition, five high-frequency approximation coefficients $D_{j}f$ and one low-frequency approximation number $A_{j}^{d}$ are obtained. The suction signal has basically been decomposed into the reconstructed signal of $A_{j}^{d}$. Since the frequency components of BCG are relatively complex and distributed in the reconstructed signals of $D_{1-3}f$, it is necessary to select a scale covering both frequency components for reconstruction in the subsequent two signal reconstruction processes. The DB5 wavelet is used to transform the original signal from the time domain to wavelet domain, and the appropriate threshold function is selected for certain nonlinear processing to eliminate the noise coefficient. Finally, according to the distribution of frequency components of the respiratory signal and BCG in each scale, the appropriate approximate wavelet coefficients are selected to reconstruct the dual signals, so as to achieve signal separation and denoising.

**Figure 9:**
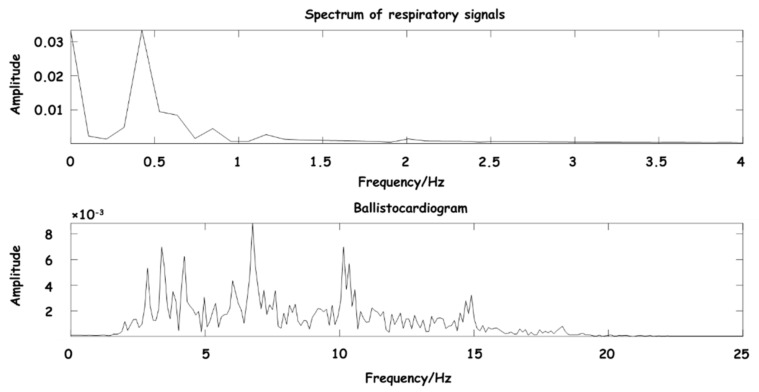
Spectrum diagram of separated signal.

In the process of testing the preprocessing algorithm, in order to ensure the effectiveness and intuitiveness of the effect, an actual signal about 10 s was randomly selected in the experiment, which was collected by the hardware acquisition device of this design. The process of signal separation and denoising in the whole experiment process was simulated, and the spectrum of the separated signal was obtained as shown in Figure 9.

As can be seen from Figure 9, the main components of BCG are between 3–15 Hz, and the main components of the respiratory signal are between 0.3–0.8 Hz, which are consistent with the expected frequency range, further indicating that the signal frequency range reconstructed by the wavelet coefficient is clear, and the separated signal has obvious waveform characteristics.

**Figure 10:**
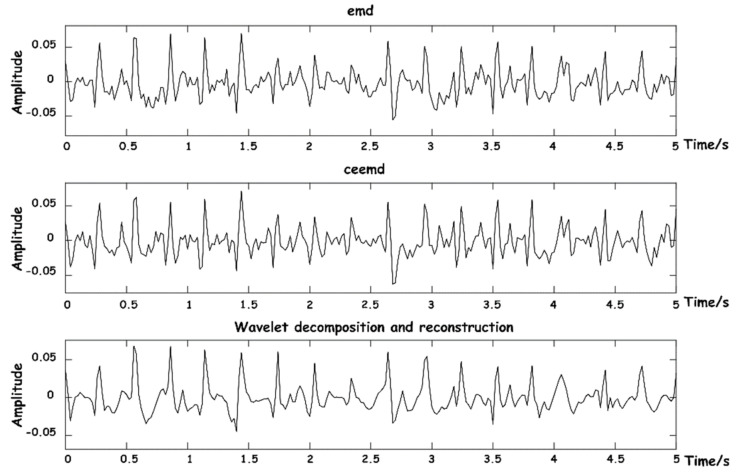
Diagram of BCG signal reconstruction effects.

A comprehensive comparison was made between EMD [22], complementary ensemble empirical mode decomposition (CEEMD) [25], and our proposed wavelet decomposition and reconstruction algorithm. The comparison of reconstruction effects of BCG is shown in Figure 10.

**Figure 11:**
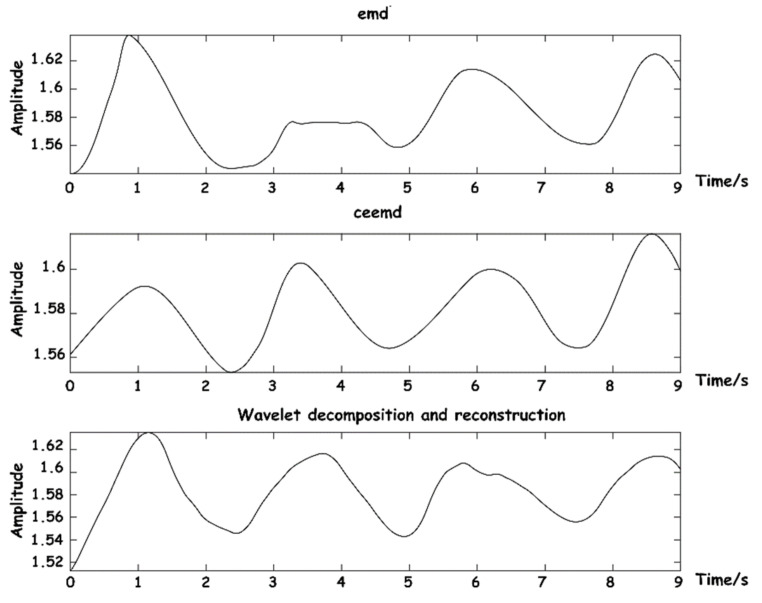
Diagram of respiratory signal reconstruction effects.

The above three methods were also used to reconstruct respiratory signals, and the results are shown in Figure 11. As can be seen from the figure, respiratory signals reconstructed by EMD are easy to be aliased into other IMF components that do not meet the energy threshold, and signal loss phenomenon easily occurs. CEEMD is an improved algorithm for the frequency aliasing phenomenon of EMD in essence, so the separation results are relatively good. The decomposition and reconstruction based on WT can restore the respiratory signal waveform with high stability.

### 3.2. Feature Parameter Extraction

After pretreatment, the reconstructed BCG signal is a relatively pure signal, and without interference noise such as baseline drift, the J-peak can be extracted. Due to the existence of a refractory period and arrhythmia, the J-peak should be corrected. A refractory period means that the average person’s heart rate is less than 300 beats per minute, and after one heartbeat occurs, no other heartbeat occurs within a certain time interval. Therefore, the specific process is as follows: first, solve the maximum value of the whole sequence, judge whether its maximum value meets the set peak threshold $U_{SH}$, and judge it as a J-peak. If a J-peak has been predetermined, other extreme points that meet the J-peak standard detected within 220 ms of the signal sequence will be ignored to avoid false detection caused by noise interference. At the same time, when the distance between an adjacent J-peak is less than 0.4 $mean_{jj}$ ($mean_{jj}$ refers to the mean of all J-peak intervals, which is temporarily determined as the mean of J-peak intervals of a breathing frame in this system), the J-peak is considered to be wrongly detected, and the J-peak with the smaller amplitude is removed if the larger amplitude is retained. When the distance between adjacent J-peaks is greater than 1.66 $mean_{jj}$, it is considered that a J-peak has been missed, and the threshold value should be adjusted to be detected again in this interval. If the J-peak is still not found, arrhythmia will be identified and the next J-peak will be determined [26]. The system defines the threshold as follows, where bias is the adaptive offset value:

Peak threshold:

(2) $$U_{SH}<0.02+bias$$

Minimum peak-to-peak interval threshold:

(3) $$D_{SH}<0.22$$

**Figure 12:**
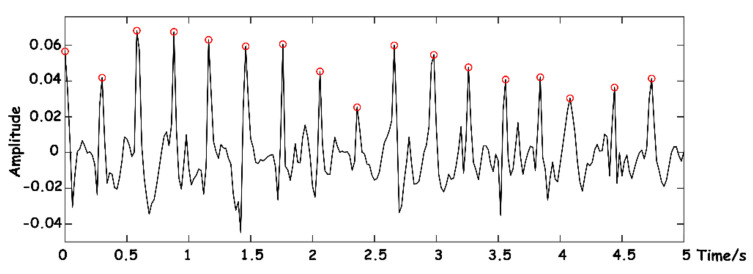
J-wave-peak extraction.

The position of a J-peak can be accurately calibrated. As shown in Figure 12, the identified J-peaks are marked with red circles.

**Figure 13:**
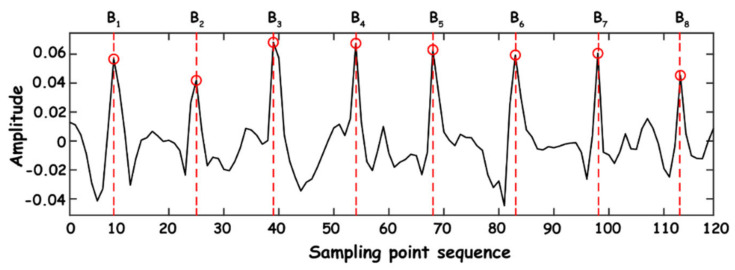
Time point extraction.

General heart rate refers to the number of beats of the heart per minute, which can be indirectly expressed by the instantaneous heart rate, and the instantaneous heart rate can be obtained by calculating the reciprocal of the time difference between BCG’s adjacent J-wave peaks. Assume that the time point of peak J of each BCG signal is $B_{n}$ in the sequence of the current breathing frame, as shown in Figure 13.

The heartbeat times of normal people, namely HR, are generally between 60 and 120 times within 1 min. In this work, the average of normal-to-normal interval (*AVNN*) index should be calculated using the mean value within a respiration wave, and its calculation expression is as follows:

(4) $$AVNN=\frac{1}{N-1}\sum_{i=1}^{N-1}\frac{F_{n}}{B_{n+1}-B_{n}}(N>1)$$

where N is the number of J-peaks of BCG in a respiratory wave, and $F_{n}$ is the sampling frequency of hardware collection equipment.

The calculation of standard deviation (SD) during successive heartbeats is mainly performed on all heartbeat intervals within each breathing frame in this work, and standard deviation of normal-to-normal intervals (*SDNN*) can be obtained:

(5) $$SDNN=\frac{1}{N-1}\overset{N-1}{\underset{i=1}{\sum }}\frac{F_{n}}{B_{n+1}-B_{n}}(N>1)$$

The coefficient of variation (*CV*) is essentially the quotient of SD and mean heart rate during successive heartbeats, which can be translated into the ratio of partial variability and is easily predicts and reflect the variation in human physiological conditions. The expression is as follows:

(6) $$CV=\frac{AVNN}{SDNN}$$

The breath wave period is the time length of a breath wave. In the process of breathing, the end of exhalation corresponds to the peak of the breath wave and the end of inspiration corresponds to the trough of the breath wave. Therefore, the breath wave period can be obtained by subtracting two breath wave peaks or by subtracting two breath wave troughs. A relatively complete breath wave was obtained in the single frame interception of signal preprocessing. In order to reduce computation, the number of sampling points of the current breath frame was used in this work and divides the sampling frequency to obtain the breath wave period.

Equation (7) is the calculation formula of the breath wave period $T_{i}$, $C_{i}$ is the number of sampling points in the current breath frame, and $F_{n}$ is the sampling frequency of hardware acquisition equipment.

(7) $$T_{i}=C_{i}/F_{n}$$

The similarity of the respiratory waveform is the calculation of the similarity between the normal and reconstructed respiratory waveform, which can directly reflect the abnormal state of respiration. After the above single frame interception and respiration reconstruction, a single respiration waveform can be obtained at this time. Since respiration intensity varies from person to person, in order to uniformly use a normal respiration waveform, it is necessary to normalize the entropy of reconstructed waveform data and then judge the degree of cross-correlation. The cross-correlation judgment formula is as follows:

(8) $$R_{f,g}\left(n\right)=\sum_{k=0}^{N}f^{*}\left(k\right)\cdot g\left(k+n\right)$$

where f is the standard respiratory wave data series, g is the respiratory wave data series obtained from actual collection and reconstruction, $R_{f,g}$ is the correlation degree value between f and g sequences, n is the delay time of correlation calculation, and n is less than the sum of the length of f and g sequences.

**Figure 14:**
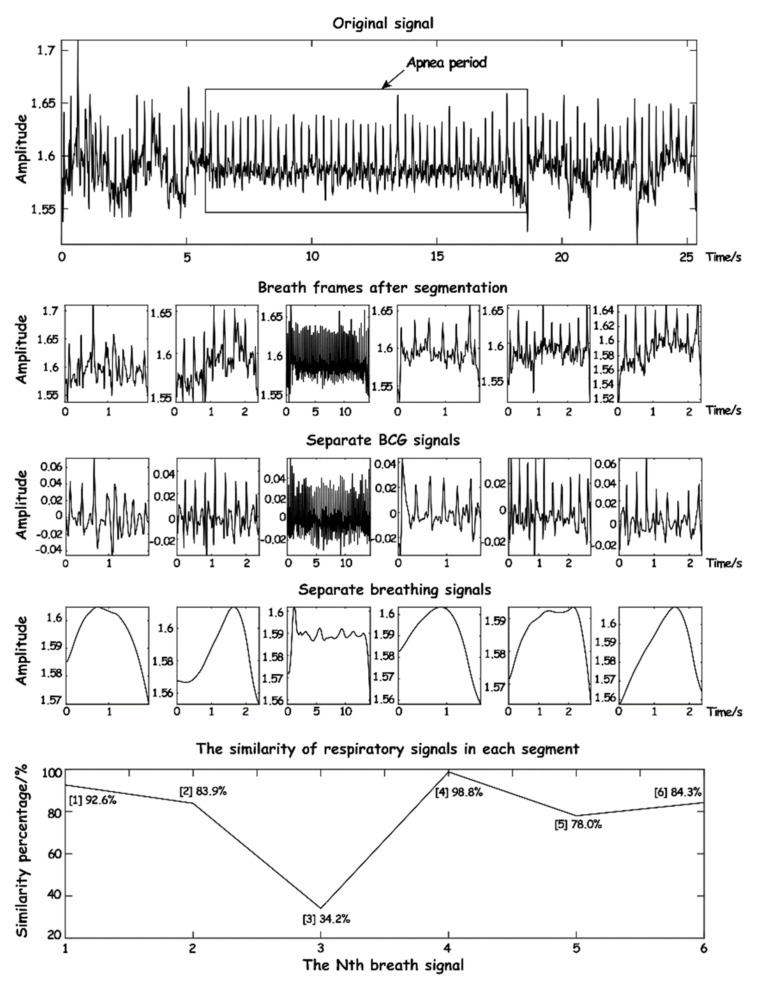
The schematic diagram of respiratory waveform similarity.

Since respiratory signals have randomness of amplitude, frequency, and time, the delay time cannot be determined by logical operation. Therefore, the maximum value of the correlation of the two sequences is used as the similarity of the two signals, and the formula is as follows:

(9) $$\xi =\frac{max\left(R_{f,g}\right)}{max\left(R_{f,f}\right)}\times 100\%$$

where $R_{f,f}$ is the autocorrelation degree value of a standard respiratory wave data series. By the calculated value of ξ, the current interception of breath and breathing normally wave similarity can be learned, in addition to the breathing waveform capture setting adaptive threshold and the entropy integral normalized processing, making the breathing waveform similarity calculation have strong adaptability. As shown in Figure 14, the signal within 25 s is processed and a similarity of the respiratory waveform is calculated.

As can be seen from Figure 14, when calculating normal respiratory signals, the similarity is over 70%, while when abnormal respiration occurs, the similarity is significantly reduced, especially when apnea occurs, where the similarity is less than 40% and lasts for a long time. It can be seen that the respiratory waveform similarity has a high sensitivity to the signal during apnea time.

### 3.3. Classification of SAHS

Using physiological signal parameters to determine sleep apnea is essentially a dichotomous problem. The logistic regression model is mainly used for dichotomous detection [27,28,29]. Compared with the existing machine learning model, the calculation is faster and simpler, and more suitable for engineering projects [30]. Therefore, this project designed a binomial logistic regression model to discriminate and classify.

In the binomial logistic regression model by the conditional probability distribution P(Y|X), according to the independent variables in which previous calculation of the signal characteristic parameters of $X={\left(x^{\left(1\right)},x^{\left(2\right)},\ldots \;\ldots x^{\left(m\right)}\right)}^{T}$ are valued as the real vector space of m dimension $R^{m}$, the dependent variable is the output variable Y values of 1 and 0. Assuming that the distribution of independent variable parameters of sleep apnea follows the property of a logistic regression model, the conditional probability distribution of the model is shown in Equations (10) and (11) [31]:

(10) $$P(Y=1|X=x)=\frac{exp\left(w\cdot x+b\right)}{1+exp\left(w\cdot x+b\right)}$$

(11) $$P(Y=0|X=x)=\frac{1}{1+exp\left(w\cdot x+b\right)}$$

where $w={\left(w_{1},w_{2},\ldots \;\ldots ,w_{m}\right)}^{T}\in R^{m}$ is the weight vector parameter and b∈R is the offset parameter. In addition, w·x represents the inner product of w and x, namely $w^{T}x$.

When uniformly classifying the weight vector parameters w and b, it can still be denoted as w. In this case, $w={\left(w_{1},w_{2},\ldots \;\ldots ,w_{m},b\right)}^{T}$ is also denoted as $X={\left(x^{\left(1\right)},x^{\left(2\right)},\ldots \;\ldots x^{\left(m\right)},1\right)}^{T}$. At this point, the binomial logistic regression model can be expressed by Equations (12) and (13) [31]:

(12) $$P(Y=1|X=x)=\frac{exp\left(w\cdot x\right)}{1+exp\left(w\cdot x\right)}$$

(13) $$P(Y=0|X=x)=\frac{1}{1+exp\left(w\cdot x\right)}$$

After using the experimental data for model parameters and determining the value vector parameter w, the actual test data on the plug type and output parameters can be obtained for the 1 s and 0 s  P(Y=1|X=x) and P(Y=0|X=x) probability. Then, according to the preset threshold v to judge the value of the output parameter Y, if $P\left(Y=1│X=x\right)\geq v$, then the output parameter Y is judged as 1, otherwise it is 0, usually the threshold v=0.5.

Maximum Likelihood Estimate (MLE) is used to solve the parameters to be estimated in the Logit regression model [32,33]. Assume that $P\left(Y=1│X=x\right)=\pi \left(x\right)$, then $P\left(Y=0│X=x\right)=1-\pi \left(x\right)$, and for n samples of the training data set, the likelihood function is shown in Equation (14):

(14) $$L\left(w\right)=\overset{n}{\underset{i=1}{\prod }}{\left[\pi \left(x_{i}\right)\right]}^{y_{i}}{\left[1-\pi \left(x_{i}\right)\right]}^{1-y_{i}}$$

The logarithmic likelihood function is shown in Equation (15):

(15) $$l\left(w\right)=ln\left(L\left(w\right)\right)=\sum_{i=1}^{n}\left[y_{i}\left(w\cdot x_{i}\right)-ln\left(1+exp\left(w\cdot x_{i}\right)\right)\right]$$

Then, the estimated parameter $\hat{w}$ can be obtained by solving the maximum value of $l\left(w\right)$. When solving $\hat{w}$, the gradient iteration method or Newton iteration method is generally adopted, and the binomial Logit regression model estimated at last is shown in Equations (16) and (17):

(16) $$P(Y=1|x)=\frac{exp\left(\hat{w}\cdot x\right)}{1+exp\left(\hat{w}\cdot x\right)}$$

(17) $$P(Y=0|x)=\frac{1}{1+exp\left(\hat{w}\cdot x\right)}$$

Assume that 800 groups of sample data are inserted into the model independent variables and the estimated parameter $\hat{w}$ can be obtained after the above iterations. Therefore, the binomial Logit regression model can be constructed.

## 4. Verification

**Figure 15:**
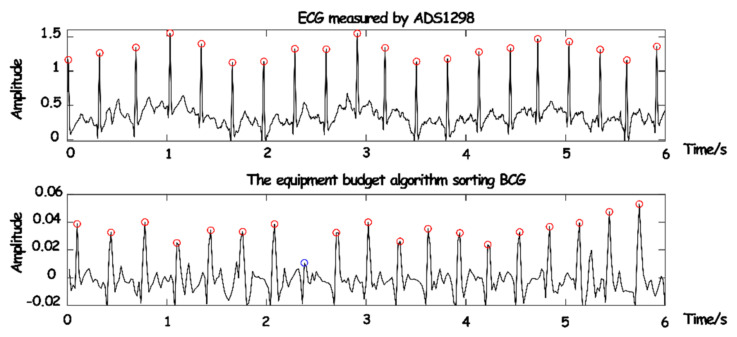
The synchronization comparison between BCG and ECG.

Before the establishment of the model, in order to verify the accuracy of the parameters obtained in the experiment, 100 groups of preprocessed breath frames were randomly selected from the experimental data, and the heartbeat times and duration of each breath frame were taken as the analytical data for verification. These two parameters had a decisive impact on the characteristic parameters of the model. The synchronous comparison between the ECG monitoring device and the actual separated BCG signals is shown in Figure 15.

As the conduction velocity of an electrical signal is much higher than that of a pressure signal, the occurrence of BCG will lag behind that of ECG. It can be seen that only one (blue circle in Figure 15) BCG signal was not detected due to partial interference or the influence of human body motion. However, the J-peak of BCG and the R-peak of ECG can be synchronized after phase shift, the overall detection rate of the BCG signal was quite high, and part of the false detection and missing detection can be removed in the subsequent equalization and depolarization.

**Figure 16:**
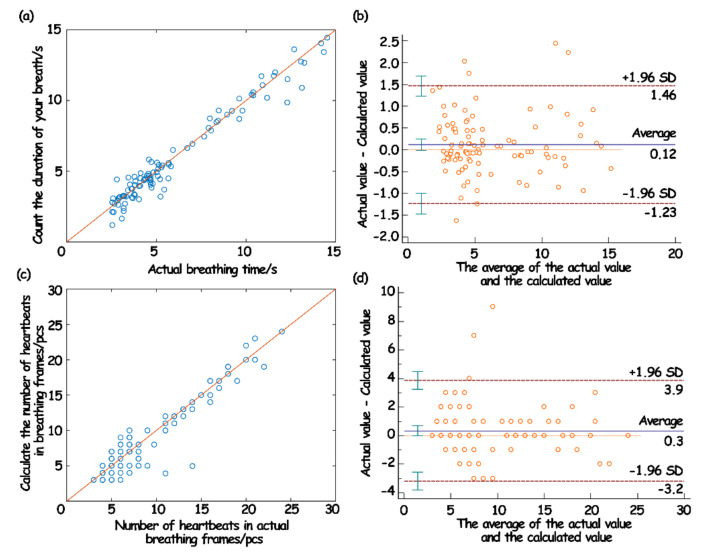
The correlation of respiratory wave duration (**a**) and Bland–Altman analysis diagram (**b**); the correlation of heartbeat number (**c**) and Bland–Altman analysis chart in breathing frame (**d**).

In this work, the Pearson correlation coefficient and Bland–Altman diagram were introduced for consistency assessment. Among them, the Pearson correlation coefficient can be a good measure of linear correlation [34,35], and the Bland–Altman diagram can intuitively show the consistency of reaction data [36,37]. The results are shown in Figure 16.

The evaluation results showed that the duration of a single respiratory wave and the number of jumps in the breathing frame measured by the sleep monitoring system had a strong correlation with the measured actual value, which indicated that the predicted value was highly linear relative to the actual value (Figure 16a,c). Moreover, in the Bland–Altman analysis, the variation in the average can be described by the SD. When the difference in the measurement results follows a normal distribution, then 95% of the difference should be between $\bar{d}-1.96SD,\bar{d}-1.96SD$, which is called the 95% limits of agreement (LoA) [38]. From the obtained results, it can be seen that 95% of the points in the breath detection fell within the 95% LoA (Figure 16b). In the heartbeat detection, 97% of the points fell within the 95% LoA (Figure 16d). Such results showed that the data collected and processed by the system in this work were consistent with the actual situation, and also showed that the physiological data parameters based on the system in this work can be used for the actual model establishment and analysis.

The test data of four volunteers were substituted into the constructed binomial Logit regression model as experimental verification data to classify apnea. The classification results are shown in Table 3.

The results showed that the identification accuracy of apnea based on Logit regression model was about 90%, which basically could identify apnea symptoms well. For the undetected data, the reason might be due to the short duration of apnea, which was at the boundary of the discriminant condition. If the fitting sample was large enough and the output parameter threshold was optimized, the classification accuracy would be greatly improved.

The research on the monitoring of sleep apnea based on mattresses is relatively limited, and the conclusions of the research are inconsistent. For example, K. Nagatomo et al. [39] compared the Nemuri SCAN (NSCAN) mattress produced by Paramount Bed Co., Ltd. with the reference standard PSG for 24 h. The results showed that although the subjective sleep evaluation of the Richards-Campbell Sleep Questionnaire was positively correlated with the parameters measured by PSG, it was not correlated with the sleep parameters measured by NSCAN, and the role of NSCAN in critical patients needed further exploration. J. Toominen et al. [40] used the commercially available household sleep monitoring device Beddit sleep Tracker (BST) to study the simultaneous sleep of PSG and BST for two consecutive nights in 10 healthy young people. This research showed that this device has poor correlation in estimating sleep continuity measurement and is not an effective device for monitoring sleep. P. Edouard et al. [36] completed one night of simultaneous PSG and Withings Sleep Analyzer (WAS) recording in a sleep clinic for 118 patients suspected of obstructive sleep apnea syndrome. The best sensitivity that WAS obtained was 88%. The authors believe that WAS can accurately detect moderate to severe sleep apnea syndrome in patients suspected of sleep apnea syndrome, which can have great clinical value. In comparison, the 90% accuracy rate presented in our study is still considerable. However, there are limitations to our study. First of all, only some important parameters were selected from the extracted signal data to determine the binary model of apnea. In fact, there are some special states in human sleep, such as turning over and snoring, which are closely related to individual diseases and may lead to misleading judgment of apnea. Secondly, more information can be mined during sleep, such as sleep stages, sleep quality assessment, etc. More accurate sleep monitoring indicators can be obtained by using more complex classification methods. Finally, although the symptoms targeted by this working system cover a large number of people, it is difficult to really extract the naturally occurring apnea signal in the actual test. Therefore, it is hoped that in the future, there will be more opportunities to truly apply it to the clinic and obtain more data to improve the model so as to promote the accuracy and practicability of this system to a wider direction.

## 5. Conclusions

In this work, we designed a mattress-type non-affecting sleep apnea monitoring system, which can extract BCG and respiratory signals from the pressure signals during sleep. In signal pretreatment, WT was used for signal separation and denoising to directly and accurately separate BCG and respiratory signals. In feature extraction, the respiratory waveform similarity index was innovatively proposed on the basis of SAHS common features. A binomial logistic regression model was used to determine the symptoms of sleep apnea in the signal segment during apnea detection. Simulation and experimental results showed that the algorithm has high availability. In this work, respiratory problems were analyzed, but there is still a lot of information in sleep that can be mined, such as sleep staging and sleep quality assessment. For this additional information, more complex classification methods, such as support vector machines and decision trees, can be used to build models so as to obtain more comprehensive analysis, which can also make the signals extracted by the system in this work applied in a wider range.

## Figures and Tables

**Table 1:** Comparison of performance between BCG and ECG.

Comparative Item	BCG (Our System)	ECG
Signal properties	Mechanical vibration signal	Electrophysiological signal
Signal accuracy	Medium	High
Channels of electrodes	One channel	Multi channels or one channel
Cost	Low	High
Form of signal acquisition equipment	Mattress	Holter monitoring, wearable devices, etc.

**Table 2:** Interval values and signal components of each part after wavelet decomposition.

Scale (2*^j^*, *j* = 1, 2, …)	Main Frequency Band (Hz)	The Signal Component
$D_{1}f$	12.5–25	Noise with a small ballistocardiogram component
$D_{2}f$	6.25–12.5	Ballistocardiogram is the main component
$D_{3}f$	3.125–6.25	Low frequency ballistocardiogram + body motion noise
$D_{4}f$	1.5625–3.125	Some body moving noise
$D_{5}f$	0.7813–1.5625	Partial body movement + partial respiration
$A_{j}^{d}$	0–0.7813	Respiratory signal and DC baseline

**Table 3:** The verification result statistics of test data of four volunteers.

Tester	Actual Apnea	The Model Identified Apnea Times	Leaving Out the Number	Percentage of Accuracy
1	5	5	0	100%
2	4	4	0	100%
3	8	7	1	87.5%
4	3	2	1	66.7%
mean	5	4.5	0.5	90%

## Data Availability

Not applicable.
